# Features which discriminate between tuberculosis and haematologic malignancy as the cause of pleural effusions with high adenosine deaminase

**DOI:** 10.1186/s12931-023-02645-6

**Published:** 2024-01-04

**Authors:** Junsu Choe, Sun Hye Shin, Kyeongman Jeon, Hee Jae Huh, Hyung-Doo Park, Byeong-Ho Jeong

**Affiliations:** 1 Division of Pulmonary and Critical Care Medicine, Department of Medicine, Samsung Medical Center, Sungkyunkwan University School of Medicine, 81 Irwon-ro, Gangnam-gu, Seoul, 06351 Republic of Korea; 2 Department of Laboratory Medicine and Genetics, Samsung Medical Center, Sungkyunkwan University School of Medicine, Seoul, Republic of Korea; 3 Department of Medical Device Management and Research, Samsung Advanced Institute for Health Sciences & Technology, Sungkyunkwan University, Seoul, Republic of Korea

**Keywords:** Adenosine deaminase, Tuberculous Pleurisy, Malignant pleural effusion, Lymphoma

## Abstract

**Background:**

Adenosine deaminase (ADA) is a useful biomarker for the diagnosis of tuberculous pleurisy (TBP). However, pleural effusions with high ADA can also be caused by other diseases, particularly hematologic malignant pleural effusion (hMPE). This study aimed to investigate the features that could differentiate TBP and hMPE in patients with pleural effusion ADA ≥ 40 IU/L.

**Methods:**

This was a retrospective observational study of patients with pleural effusion ADA ≥ 40 IU/L, conducted at a Korean tertiary referral hospital with an intermediate tuberculosis burden between January 2010 and December 2017. Multivariable logistic regression analyses were performed to investigate the features associated with TBP and hMPE, respectively.

**Results:**

Among 1134 patients with ADA ≥ 40 IU/L, 375 (33.1%) and 85 (7.5%) were diagnosed with TBP and hMPE, respectively. TBP and hMPE accounted for 59% (257/433) and 6% (27/433) in patients with ADA between 70 and 150 IU/L, respectively. However, in patients with ADA ≥ 150 IU/L, they accounted for 7% (9/123) and 19% (23/123), respectively. When ADA between 40 and 70 IU/L was the reference category, ADA between 70 and 150 IU/L was independently associated with TBP (adjusted odds ratio [aOR], 3.11; 95% confidence interval [CI], 1.95–4.95; *P* < 0.001). ADA ≥ 150 IU/L was negatively associated with TBP (aOR, 0.35; 95% CI, 0.14–0.90; *P* = 0.029) and positively associated with hMPE (aOR, 13.21; 95% CI, 5.67–30.79; *P* < 0.001). In addition, TBP was independently associated with lymphocytes ≥ 35% and a lactate dehydrogenase (LD)/ADA ratio < 18 in pleural effusion. hMPE was independently associated with pleural polymorphonuclear neutrophils < 50%, thrombocytopenia, and higher serum LD. A combination of lymphocytes ≥ 35%, LD/ADA < 18, and ADA < 150 IU/L demonstrated a sensitivity of 0.824 and specificity of 0.937 for predicting TBP.

**Conclusion:**

In patients with very high levels of pleural effusion ADA, hMPE should be considered. Several features in pleural effusion and serum may help to more effectively differentiate TBP from hMPE.

**Supplementary Information:**

The online version contains supplementary material available at 10.1186/s12931-023-02645-6.

## Introduction

According to two national surveys conducted in the United States and China, extrapulmonary tuberculosis accounted for 18–25% of all tuberculosis cases, and tuberculous pleurisy (TBP) is one of the most common presentations of extrapulmonary tuberculosis, accounting for approximately 20–35% [1, 2]. In addition, TBP is the main cause of pleural effusion in many countries [3]. The gold standard for the diagnosis of TBP is the microbiological confirmation of *Mycobacterium tuberculosis* in pleural effusion or histopathological confirmation in pleura. However, the sensitivities of culture, nucleic acid amplification tests for pleural effusion, and histopathological examination for pleura are as low as 30–60% to diagnose TBP [3–5].

Adenosine deaminase (ADA) in pleural effusion is a widely accepted diagnostic marker for TBP. The most commonly used ADA cut-off value of 40 international units (IU)/L shows excellent sensitivity and specificity of 0.93 and 0.90, respectively [6]. However, even if the ADA level of pleural effusion exceeds 40 IU/L, there is a possibility for a disease other than TBP including parapneumonic effusion (PPE) and malignant pleural effusion (MPE) [7]. Although higher ADA levels have traditionally been associated with a higher likelihood of TBP [8], recent studies have reported that extremely high ADA levels may indicate a low probability of TBP and that the possibility of lymphoma should be considered in diagnosis [9, 10].

Delayed diagnosis and misdiagnosis for TBP and MPE can have detrimental effects on treatment outcomes. It is usually not difficult to differentiate PPE and solid MPE (sMPE) because most patients with PPE and sMPE have a high proportion of pleural polymorphonuclear neutrophil (PMN) and primary mass or pleural nodularity on imaging tests, respectively. However, hematologic MPE (hMPE) should be suspected based on only pleural effusion and blood analysis findings, so misdiagnosing hMPE as TBP may occur [11]. Therefore, this study aimed to investigate the factors that could differentiate TBP and hMPE in patients with pleural effusion ADA ≥ 40 IU/L in a hospital in Korea with an intermediate tuberculosis burden.

## Methods

### Study population

This was a retrospective observational study of patients with pleural effusion ADA ≥ 40 IU/L. We retrospectively reviewed the patients who underwent diagnostic thoracentesis between January 2010 and December 2017 at Samsung Medical Center, a university-affiliated, tertiary referral hospital with 2000 beds in Seoul, Korea. If a patient had undergone more than one thoracentesis, only the results of the first procedure were analyzed. Patients with missing values ​​in pleural effusion analysis, younger than 18 years of age, lost to follow-up, or with thoracentesis after treatment initiation were excluded. This study was approved by the Samsung Medical Center Institutional Review Board (SMC IRB no. 2020-02-150) to review and publish information acquired from patient records. The requirement for informed consent was waived by SMC IRB because of the observational nature of the study. Patient information was de-identified and anonymized prior to the analysis.

### Diagnostic criteria

PPE was defined as any exudative effusion associated with bacterial pneumonia, lung abscess, or bronchiectasis. Empyema was diagnosed based on the presence of thick, purulent-appearing pleural effusion or a positive bacterial culture [12]. PPE was defined as complicated if it had a pH < 7.2, glucose < 60 mg/dL, or required drainage; otherwise, it was considered an uncomplicated PPE. Chronic empyema was defined as empyema lasting more than 4 weeks with thickened visceral and parietal fibrin peels that was not thought to be effusion associated with pneumonia, lung abscess, or bronchiectasis, which is the definition of PPE [13].

Definite TBP was diagnosed by (1) confirming the presence of *Mycobacterium tuberculosis* through growth in culture or nucleic acid amplification test using pleural effusion, (2) identifying granulomas (with or without caseous necrosis) in a pleural biopsy, excluding other granulomatous diseases, or (3) positive sputum culture for *M. tuberculosis* with improvement of pleural effusion after anti-tuberculous treatment [4]. Suspected TBP was defined as clinically suspected cases of TBP where pleural effusion was resolved with anti-tuberculous treatment [14]. Patients with suspected TBP who were followed up for less than a year were excluded from the study [15].

The diagnosis of MPE was based on positive pleural fluid cytology or pleural tissue histology, or evidence of malignancy elsewhere with radiological evidence of metastasis, after exclusion of alternative causes of the effusion [16]. The diagnosis of another cause of pleural effusions was based on clinical presentation, results of the appropriate diagnostic tests, and the patient’s response to treatment.

### Statistical analysis

Data are reported as medians and interquartile ranges (IQR) for continuous variables and as numbers (percentages) for categorical variables. Data were compared using the Kruskal-Wallis test for continuous variables and the Chi-square or Fisher’s exact test for categorical variables. The Bonferroni correction was used to determine the significance of multiple comparisons.

A logistic regression analysis was performed to identify associated variables for the diagnosis of TBP and hMPE. We regarded variables significant at a value of 0.20 in univariable analysis as candidates for a multivariable regression model. To avoid multicollinearity, the multivariate analysis included either polymorphonuclear neutrophils (PMN) or lymphocytes. Continuous variables were converted to categorical variables using the highest Youden’s index or clinically meaningful cut-off values in logistic regression analysis to facilitate clinical interpretation. Recursive partitioning analysis was employed to assess all potential feature combinations in identifying TBP or hMPE [17]. After each partitioning, this process was applied iteratively to the subgroups to explore combinations with high accuracy for identifying TBP or hMPE. A simple combination for clinical application was finally presented. The recursive partitioning analysis was conducted using a tree-based methodology. All tests were two-tailed, and a *P*-value < 0.05 was regarded as statistically significant. All analyses were performed using the SPSS software (IBM SPSS Statistics ver. 27, Armonk, NY, USA) and R software version 3.6.3 (http://www.R-project.org).

## Results

**Fig. 1:**
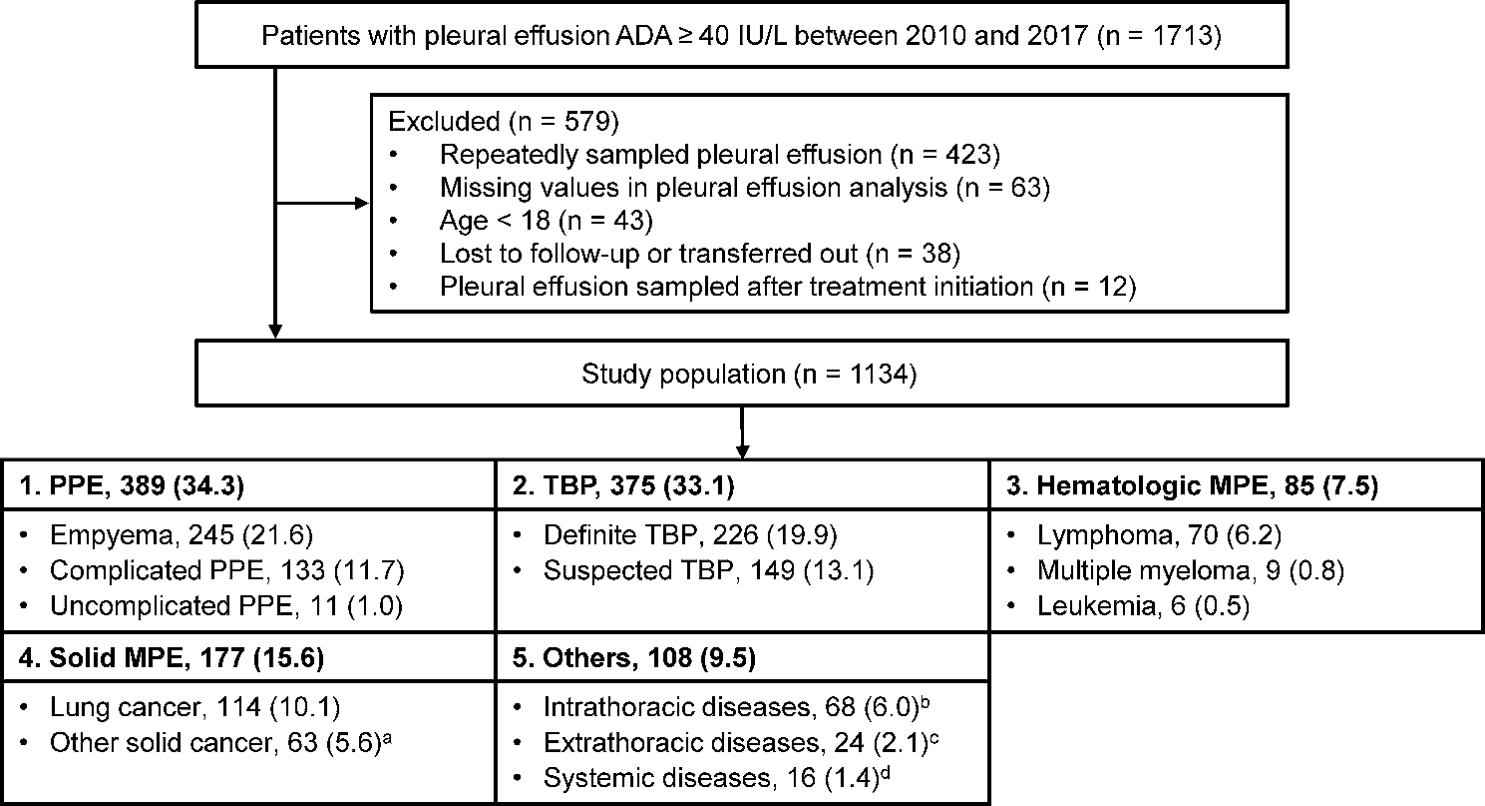
Flow chart and causes of pleural effusions with ADA ≥ 40 IU/L (n = 1134). Data are reported as numbers (%). ^a^Mesothelioma (n = 9), gastric cancer (n = 9), renal cell carcinoma (n = 8), hepatocellular carcinoma (n = 6), thymic carcinoma (n = 5), breast cancer (n = 4), cholangiocarcinoma (n = 3), pancreas cancer (n = 3), ovary cancer (n = 3), sarcoma (n = 3), thyroid cancer (n = 2), melanoma (n = 1), hypopharynx cancer (n = 1), esophageal cancer (n = 1), colon cancer (n = 1), cervix cancer (n = 1), dysgerminoma (n = 1), ampullary cancer (n = 1), and adenocarcinoma of unknown origin (n = 1). ^b^Post-thoracic surgery (n = 24), hemothorax (n = 10), chronic empyema (n = 8), pneumothorax (n = 7), interstitial lung diseases (n = 7), non-tuberculous mycobacterial lung disease (n = 4), chylothorax (n = 3), congestive heart failure (n = 2), pulmonary thromboembolism (n = 1), esophageal perforation (n = 1), and pericarditis (n = 1). ^c^Peritonitis (n = 13), liver abscess (n = 5), cholecystitis (n = 3), transcatheter arterial chemoembolization for hepatocellular carcinoma (n = 2), and post-abdominal surgery (n = 1). ^d^Parasite (n = 6), systemic lupus erythematosus (n = 4), rheumatoid arthritis (n = 3), adult-onset Still’s disease (n = 1), mixed connective tissue disease (n = 1), and hemophagocytic lymphohistiocytosis (n = 1). ADA = adenosine deaminase, MPE = malignant pleural effusion, PPE = parapneumonic effusion, TBP = tuberculous pleurisy

During the study period, 1713 patients with pleural effusion ADA ≥ 40 IU/L were identified. Of these, 579 were excluded because of repeatedly sampled pleural effusion (n = 423), missing values in pleural effusion analysis (n = 63), pediatric patients (n = 43), lost to follow-up or transferred out (n = 38), and pleural effusions sampled after treatment initiation (n = 12). Finally, 1134 patients with pleural effusion ADA ≥ 40 IU/L were included in this study (Fig. 1). Among the 1134 patients, 389 (34.3%) were diagnosed with PPE, 375 (33.1%) with TBP, 85 (7.5%) with hMPE, 177 (15.6%) with sMPE, and 108 (9.5%) with another diagnosis.

### Clinical and laboratory characteristics

Comparisons of clinical and laboratory characteristics of PPE, TBP, and hMPE are listed in Table 1. Patients with PPE were significantly more likely to be male and older. The predominant cells in pleural effusion were PMN (median 85%) in PPE, lymphocytes (median 71%) in TBP, and others (median 69%) in hMPE. Patients with PPE had lower glucose (median 20 mg/dL) in their pleural effusion and higher white blood cells (WBC) (median 12,400/µL) and segmented neutrophils (82%) in their blood than those with TBP and hMPE. Compared to patients with PPE and hMPE, those with TBP were more likely to have higher pleural protein (5.0 g/dL), higher serum protein (6.8 g/dL), and lower pleural lactate dehydrogenase (LD)/ADA (7.9). Patients with PPE had higher proportions of solid cancer (36%) and diabetes (27%) than those with TBP and hMPE. Fifty-two (61%) patients with hMPE had hematological malignancies prior to thoracentesis and the remaining patients were diagnosed with hematological malignancies along with pleural effusion. Detailed information regarding the clinical and laboratory characteristics of patients with hMPE, sMPE, and other effusions are summarized in Supplement Tables 1 and 2. Patients with lymphoma had higher ADA levels in their pleural effusion (94 vs. 52 IU/L, *P* = 0.010) than those with leukemia or multiple myeloma. However, there was no difference in pleural LD/ADA ratio (17.1 vs. 20.0, *P* = 0.327).

**Table 1:** Comparison of pleural effusion with ADA > 40 IU/L

Variables	PPE (n = 389)	TBP (n = 375)	hMPE (n = 85)	*P value*
Age, years	63 (53–71)	58 (39–73)	55 (36–66)	< 0.001^a,c^
Male	316 (81)	243 (65)	57 (67)	< 0.001^a,c^
Pleural effusion				
PMN (%)	85 (73–92)	6 (2–17)	3 (1–12)	< 0.001^a,b,c^
Lymphocytes (%)	4 (1–9)	71 (50–84)	21 (10–48)	< 0.001^a,b,c^
Eosinophils (%)	0 (0–0)	0 (0–0)	0 (0–0)	< 0.001^a^
Others (%)	9 (5–18)	17 (10–27)	69 (35–87)	< 0.001^a,b,c^
pH	7.3 (7.2–7.4)	7.3 (7.3–7.4)	7.4 (7.3–7.4)	< 0.001^a,c^
Glucose (mg/dL)	20 (3–89)	95 (74–118)	93 (47–115)	< 0.001^a,c^
Protein (g/dL)	3.9 (3.0–4.8)	5.0 (4.5–5.3)	3.6 (2.9–4.6)	< 0.001^a,b^
LD (IU/L)	3421 (1878–8035)	671 (447–1023)	1803 (1037–3519)	< 0.001^a,b,c^
ADA (IU/L)	67 (48–121)	84 (65–105)	76 (53–171)	< 0.001^a^
LD/ADA	53.0 (32.4–76.7)	7.9 (5.6–11.4)	17.8 (11.0–29.1)	< 0.001^a,b,c^
Whole blood				
WBC (×10³/µL)	12.4 (9.0–16.3)	6.1 (5.1–7.7)	6.8 (2.9–9.9)	< 0.001^a,c^
Segmented neutrophils (%)	82 (76–87)	68 (62–74)	71 (53–81)	< 0.001^a,c^
Platelets (×10³/µL)	265 (152–363)	282 (227–347)	139 (61–265)	< 0.001^a,b,c^
Protein (g/dL)	5.9 (5.3–6.6)	6.8 (6.2–7.2)	5.6 (4.9–6.5)	< 0.001^a,b^
LD (IU/L)	488 (392–702)	462 (391–546)	1114 (612–1672)	< 0.001^a,b,c^
Comorbidities				
Solid cancer	140 (36)	50 (13)	2 (2)	< 0.001^a,b,c^
Diabetes mellitus	104 (27)	57 (15)	13 (15)	< 0.001^a^
History of tuberculosis	37 (10)	37 (10)	7 (8)	0.898
Hematologic malignancy	16 (4)	15 (4)	52 (61)^d^	< 0.001^b,c^
Heart failure	21 (5)	7 (2)	3 (4)	0.034^a^
Liver cirrhosis	22 (6)	15 (4)	1 (1)	0.163
Renal replacement therapy	16 (4)	6 (2)	1 (1)	0.067
Connective tissue disease	10 (3)	11 (3)	0 (0)	0.287
Transplantation	10 (3)	13 (3)	1 (1)	0.474

Data are reported as median (interquartile range) and number (%)

^a,b,c^ indicate significant differences (p < 0.017) between PPE and TBP, TBP and hMPE, and PPE and hMPE, respectively

^d^At the time of diagnosis of pleural effusion, 52 patients had already been diagnosed with hematologic malignancies. The remaining 33 patients were diagnosed with hematologic malignancy along with their MPE.

ADA = adenosine deaminase, hMPE = hematologic malignant pleural effusion, IU = international unit, LD = lactate dehydrogenase, PMN = polymorphonuclear neutrophil, PPE = parapneumonic effusion, TBP = tuberculous pleurisy, WBC = white blood cells

### Causes of pleural effusion across ADA levels

**Fig. 2:**
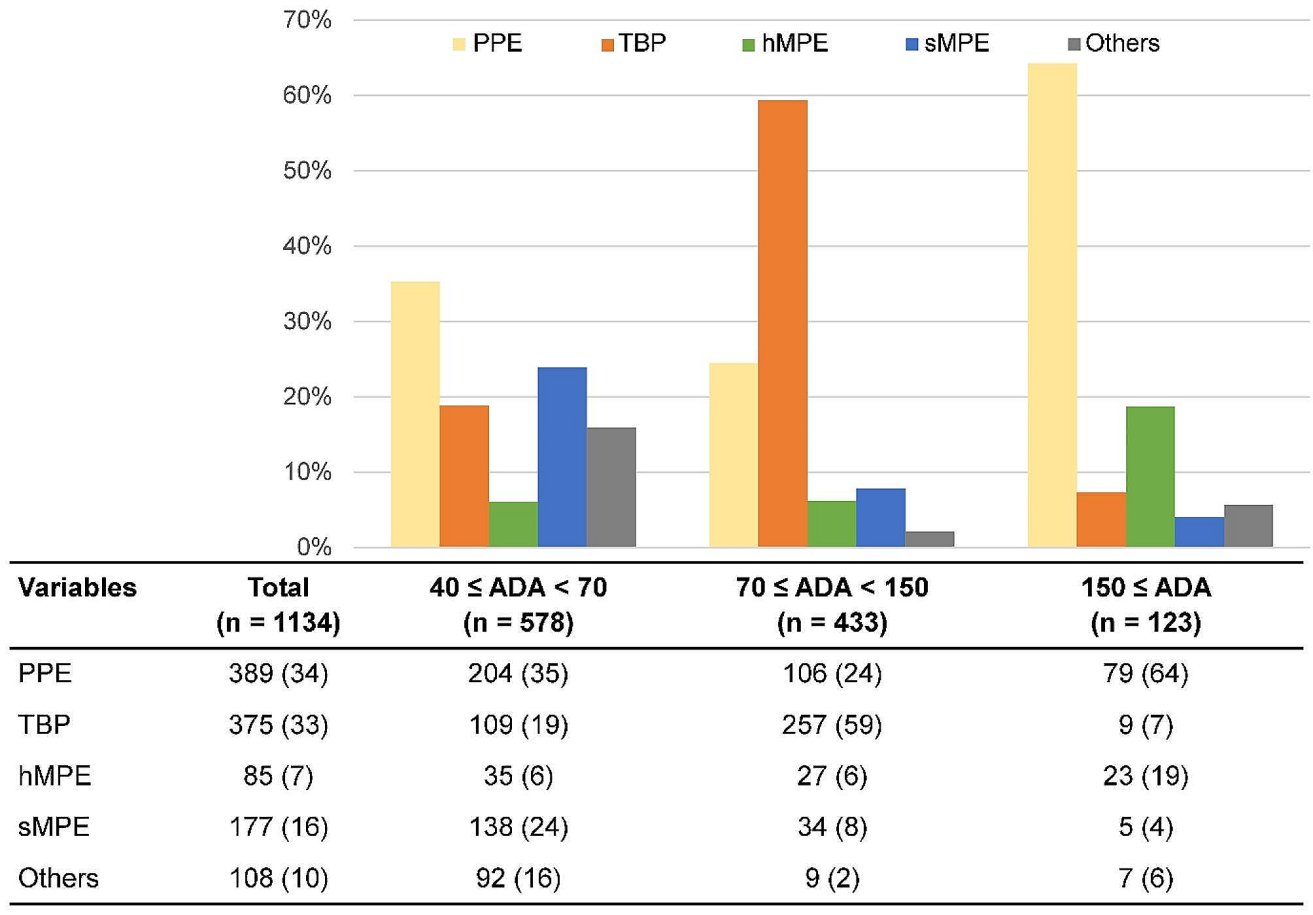
Causes of pleural effusion according to ADA level. Data are reported as numbers (%). ADA = adenosine deaminase, hMPE = hematologic malignant pleural effusion, PPE = parapneumonic effusion, sMPE = solid malignant pleural effusion, TBP = tuberculous pleurisy

The ADA levels were divided into three categories; the causes of pleural effusion for each category are shown in Fig. 2. In cases with ADA ≥ 40 IU/L and < 70 IU/L, the most common cause of pleural effusion was PPE (35%), followed by sMPE (24%) and TBP (19%). However, in pleural effusions with ADA ≥ 70 IU/L and < 150 IU/L, more than half were TBP (59%). For 123 patients with pleural effusion ADA ≥ 150 IU/L, the most common cause of pleural effusion was PPE (64%), with hMPE (19%) being more common than TBP (7%).

Of 85 patients with hMPE and 177 patients with sMPE, 8 (9%) and 5 (3%) were misdiagnosed as having TBP, resulting in delayed appropriate treatment. Of 23 patients with hMPE with ADA ≥ 150 IU/L, 4 (17%) were misdiagnosed as having TBP. In particular, of 123 patients with pleural effusion ADA ≥ 150 IU/L, only 11 patients had pleural lymphocytes ≥ 50%, which could be suspected of TBP. However, only 5/11 (45%) were finally diagnosed with TBP (5/11 [45%] = hMPE, 1/11 [9%] = sMPE).

### Features associated with TBP and hMPE

Univariable and multivariable analyses with logistic regression for variables associated with TBP are shown in Table 2. When ADA between 40 and 70 IU/L is the reference category, ADA between 70 and 150 IU/L was independently associated with TBP (adjusted odds ratio [aOR], 3.11; 95% confidence interval [CI], 1.95–4.95; *P* < 0.001), while ADA ≥ 150 IU/L was negatively associated with TBP (aOR, 0.35; 95% CI, 0.14–0.90; *P* = 0.029). Compared to pleural lymphocytes < 35%, 35–70% was independently associated with TBP (aOR, 6.67; 95% CI, 3.92–11.33; *P* < 0.001), increasing substantially at 70% or higher (aOR, 13.79; 95% CI, 7.74–24.56; *P* < 0.001). The diagnosis of TBP was also independently associated with pleural eosinophils < 10% (aOR, 4.61; 95% CI, 1.16–18.36; *P* = 0.030), pleural LD/ADA ratio < 18 (aOR, 14.00; 95% CI, 8.51–23.03; *P* < 0.001), WBC < 10,000/µL (aOR, 3.41; 95% CI, 1.92–6.03; *P* < 0.001), and platelets ≥ 150,000/µL (aOR, 2.45; 95% CI, 1.34–4.46; *P* = 0.003).

**Table 2:** Univariable and multivariable analyses with logistic regression models for variables associated with TBP in pleural effusion with ADA ≥ 40 IU/L

Variables	Univariable		Multivariable	
	**OR (95% CI)**	*P value*	**Adjusted OR (95% CI)**	*P value*
Age ≥ 65 years	1.04 (0.81–1.34)	0.743		
Pleural effusion				
ADA, IU/L				
40 ≤ ADA < 70	Reference		Reference	
70 ≤ ADA < 150	6.28 (4.73–8.34)	< 0.001	3.11 (1.95–4.95)	< 0.001
ADA ≥ 150	0.34 (0.17–0.69)	0.003	0.35 (0.14–0.90)	0.029
Lymphocytes, %				
Lymphocytes < 35	Reference		Reference	
35 ≤ Lymphocytes < 70	19.38 (12.90–29.11)	< 0.001	6.67 (3.92–11.33)	< 0.001
Lymphocytes ≥ 70	78.03 (49.06–124.11)	< 0.001	13.79 (7.74–24.56)	< 0.001
Eosinophils < 10%	4.75 (1.68–13.44)	0.003	4.61 (1.16–18.36)	0.030
pH < 7.2	0.26 (0.14–0.48)	< 0.001		
Glucose < 60 mg/dL	0.15 (0.11–0.21)	< 0.001		
Protein ≥ 2/3× serum ULN	1.72 (1.29–2.29)	0.030		
LD/ADA < 18	44.68 (29.70–67.22)	< 0.001	14.00 (8.51–23.03)	< 0.001
Serum				
WBC < 10,000/µL	10.51 (7.07–15.62)	< 0.001	3.41 (1.92–6.03)	< 0.001
Platelets ≥ 150,000/µL	3.41 (2.29–5.09)	< 0.001	2.45 (1.34–4.46)	0.003
Protein < serum LLN	3.90 (2.85–5.33)	< 0.001		
LD ≥ 2× serum ULN	0.75 (0.56–0.99)	0.043		

To avoid multicollinearity, this analysis included a proportion of pleural lymphocytes (%) only without that of pleural polymorphonuclear neutrophils

Continuous variables were converted to categorical variables using the highest Youden’s index or clinically meaningful cut-off values in logistic regression analysis to facilitate clinical interpretation

ADA = adenosine deaminase, CI = confidence interval, IU = international unit, LD = lactate dehydrogenase, LLN = lower limit of normal, OR = odds ratio, PMN = polymorphonuclear neutrophil, TBP = tuberculous pleurisy, ULN = upper limit of normal, WBC = white blood cells

Univariable and multivariable analyses with logistic regression for variables associated with hMPE are shown in Table 3. When ADA between 40 and 70 IU/L is the reference category, ADA between 70 and 150 IU/L did not show a significant association with hMPE (aOR, 0.91; 95% CI, 0.48–1.73; *P* = 0.782); however, ADA ≥ 150 IU/L was highly associated with hMPE (aOR, 13.21; 95% CI, 5.67–30.79; *P* < 0.001). Pleural PMN < 50% (aOR, 42.02; 95% CI, 11.78–149.92; *P* < 0.001), thrombocytopenia < 150,000/µL (aOR, 5.92; 95% CI, 3.28–10.68; *P* < 0.001), and serum LD ≥ 2× serum upper limit of normal (aOR, 3.88; 95% CI, 1.56–9.62; *P* = 0.003) were independently associated with hMPE.

**Table 3:** Univariable and multivariable analyses with logistic regression models for variables associated with hematologic malignant pleural effusions in pleural effusion with ADA ≥ 40 IU/L

Variables	Univariable		Multivariable	
	**OR (CI 95%)**	***P value***	**Adjusted OR (CI 95%)**	***P value***
Age ≥ 65 years	0.56 (0.34–0.91)	0.019		
Pleural effusion				
ADA, IU/L				
40 ≤ ADA < 70	Reference		Reference	
70 ≤ ADA < 150	1.03 (0.61–1.73)	0.906	0.91 (0.48–1.73)	0.782
ADA ≥ 150	3.57 (2.02–6.30)	< 0.001	13.21 (5.67–30.79)	< 0.001
Lymphocytes, %				
Lymphocytes < 35	Reference			
35 ≤ Lymphocytes < 70	0.96 (0.55–1.66)	0.870		
Lymphocytes ≥ 70	0.52 (0.27–1.01)	0.054		
PMN < 50%	22.36 (7.02–71.22)	< 0.001	42.02 (11.78–149.92)	< 0.001
Eosinophils ≥ 10%	0.97 (0.30–3.22)	0.965		
pH < 7.2	0.79 (0.34–1.86)	0.590		
Glucose < 60 mg/dL	0.60 (0.37–0.98)	0.042		
Protein ≥ 2/3× serum ULN	0.16 (0.06–0.43)	< 0.001		
LD/ADA < 18	1.35 (0.87–2.11)	0.181		
Serum				
WBC ≥ 10,000/µL	0.56 (0.33–0.94)	0.027		
Platelets < 150,000/µL	5.26 (3.30–8.41)	< 0.001	5.92 (3.28–10.68)	< 0.001
Protein < serum LLN	0.29 (0.18–0.47)	< 0.001		
LD ≥ 2× serum ULN	6.35 (2.72–14.80)	< 0.001	3.88 (1.56–9.62)	0.003

Continuous variables were converted to categorical variables using the highest Youden’s index or clinically meaningful cut-off values in logistic regression analysis to facilitate clinical interpretation

ADA = adenosine deaminase, CI = confidence interval, IU = international unit, LD = lactate dehydrogenase, LLN = lower limit of normal, OR = odds ratio, PMN = polymorphonuclear neutrophil, ULN = upper limit of normal, WBC = white blood cells

Univariable and multivariable analyses with logistic regression for variables associated with PPE and sMPE are shown in Supplement Tables 3 and 4.

### Diagnostic performances of combinations of features

The diagnostic performance of features obtained from the multivariate analysis for identifying TBP and hMPE in pleural effusions with ADA ≥ 40 IU/L was assessed (Table 4). For TBP, lymphocytes of ≥ 35% and ≥ 50% demonstrated sensitivities of 0.885 and 0.755, specificities of 0.823 and 0.887, negative predictive values (NPV) of 0.936 and 0.880, positive predictive values (PPV) of 0.712 and 0.767, and accuracies of 0.843 and 0.843, respectively. A combination of lymphocytes ≥ 35%, LD/ADA < 18, and ADA < 150 demonstrated sensitivity, specificity, NPV, PPV, and accuracy of 0.824, 0.937, 0.915, 0.866, and 0.900, respectively. For predicting hematologic MPE in pleural effusion with ADA ≥ 150, a combination of PMN < 50% and serum LD ≥ 2 × serum ULN presented corresponding values of 0870, 0.857, 0.952, 0.667, and 0.860.

**Table 4:** The diagnostic performances of selected features for tuberculous pleurisy and hematologic malignant pleural effusion in pleural effusions with ADA ≥ 40 IU/L

Categories	Sensitivity (CI 95%)	Specificity (CI 95%)	NPV (CI 95%)	PPV (CI 95%)	Accuracy (CI 95%)
***Tuberculous pleurisy***					
Lymphocyte ≥ 50%	0.755 (0.708–0.797)	0.887 (0.862–0.908)	0.880 (0.855–0.902)	0.767 (0.720–0.809)	0.843 (0.821–0.864)
Lymphocyte ≥ 35%	0.885 (0.849–0.916)	0.823 (0.794–0.850)	0.936 (0.914–0.953)	0.712 (0.669–0.753)	0.843 (0.821–0.865)
LD/ADA < 18	0.917 (0.885–0.943)	0.801 (0.771–0.829)	0.951 (0.932–0.967)	0.695 (0.652–0.735)	0.840 (0.817–0.860)
Lymphocyte ≥ 35% and LD/ADA < 18	0.837 (0.796–0.873)	0.926 (0.905–0.944)	0.920 (0.899–0.938)	0.849 (0.808–0.884)	0.897 (0.878–0.914)
Lymphocyte ≥ 35% and LD/ADA < 18 and ADA < 150	0.824 (0.782–0.861)	0.937 (0.917–0.953)	0.915 (0.893–0.934)	0.866 (0.826–0.899)	0.900 (0.880–0.916)
***Hematologic MPE in pleural effusion with ADA ≥ 150***					
PMN < 50%	0.913 (0.720–0.989)	0.850 (0.765–0.914)	0.977 (0.919–0.997)	0.583 (0.408–0.745)	0.862 (0.788–0.917)
PMN < 50% and serum LD ≥ 2× serum ULN	0.870 (0.664–0.972)	0.857 (0.753–0.929)	0.952 (0.867–0.990)	0.667 (0.472–0.827)	0.860 (0.822–0.941)

Continuous variables were converted to categorical variables using the highest Youden’s index or clinically meaningful cut-off values in logistic regression analysis to facilitate clinical interpretation

ADA = adenosine deaminase, CI = confidence interval, LD = lactate dehydrogenase, MPE = malignant pleural effusion, NPV = negative predictive value, PMN = polymorphonuclear neutrophil, PPV = positive predictive value, ULN = upper limit of normal

## Discussion

The objective of this study was to identify features associated with TBP and hMPE in patients with pleural effusion ADA ≥ 40 IU/L. In our study, features associated with TBP were pleural ADA between 70 and 150 IU/L (inversely, negative association with ADA ≥ 150), lymphocytes ≥ 35%, eosinophils < 10%, LD/ADA ratio < 18, absence of leukocytosis, and absence of thrombocytopenia. Features associated with hMPE included pleural ADA ≥ 150 IU/L, PMN < 50%, thrombocytopenia, and high serum LD. To the best of our knowledge, this is the largest study to analyze patients with high pleural effusion ADA and the first to present features associated with hMPE.

ADA, an enzyme involved in the purine pathway of DNA metabolism, is ubiquitously distributed in most human tissues. It has an essential function in lymphoid cell differentiation as well as the maturation of monocytes into macrophages, and its activity is highest in lymphoid tissues [8]. The relative abundance of T lymphocytes, which orchestrate the inflammatory response to tuberculosis, induces an elevation in ADA levels, particularly noticeable in cases of TBP. Nonetheless, pleural lymphocytic infiltration secondary to lymphoma also leads to an increase in pleural effusion ADA levels, with 25–56% of lymphomatous pleural effusions demonstrating ADA levels above the standard TB cutoff [9, 18, 19]. ADA levels exceeding 40 IU/L are not exclusive to lymphoma, as similar elevations can also be observed in pleural effusion secondary to leukemia or multiple myeloma. In a previous report including 47 cases of non-tuberculous lymphocytic exudate with ADA > 35 IU/L, two cases were caused by lymphoid leukemia and one by multiple myeloma [9]. One notable study reported a median ADA level of 37 IU/L (range: 2.8–117.8 IU/L) in a cohort of 19 patients with pleural effusion attributed to multiple myeloma [20]. Cytologic examination of lymphomatous pleural effusion has the lowest diagnostic yield of less than 20% among the various causes of MPE [21]. As a consequence, there is a significant risk of lymphoma patients being misdiagnosed with TBP, as exemplified in this and prior reports, leading to delays in the initiation of appropriate treatment strategies [22]. Therefore, it is of paramount importance to identify features associated with hMPE in cases of pleural effusions demonstrating high ADA to facilitate timely and accurate diagnosis.

Thrombocytopenia, identified as a feature of hMPE in our study, is a well-established clinical manifestation associated with hematologic malignancies. Several mechanisms can contribute to its occurrence, such as involvement of the bone marrow, splenomegaly, immune thrombocytopenia, and chemotherapy, among others [23]. LD, another independent indicator identified in our research, catalyzes the interconversion of lactate and pyruvate under hypoxic conditions, providing an environment conducive to the rapid proliferation of malignant cells. A surge in serum LD levels is typically observed in hematologic malignancies, and is often indicative of high tumor burden and aggressive disease, thereby making it a robust poor prognostic indicator [24]. Accordingly, serum LD has been previously reported as a discriminating marker that differentiates lymphoma from bacterial infections and tuberculosis [7, 10, 25]. In our study, serum LD was reaffirmed as an independent feature of hMPE, further emphasizing its potential role in diagnostic algorithms.

Our study notably elucidates that very high ADA levels serve as an independent indicator for hMPE. Previous studies showed no significant difference in ADA levels between TBP and hMPE [7, 10]. However, hMPE is more frequently associated with very high pleural ADA than TBP. In a report by Porcel and colleagues in Spain, among 22 patients presenting with pleural ADA levels exceeding 250 IU/L, 19 were diagnosed with empyema, 3 with lymphoma, and none with TBP [9]. Our research design was deliberately tailored to examine ADA ≥ 40 IU/L, which is the commonly recognized diagnostic cutoff for TBP. By limiting our study population to those with high ADA levels, we were able to evaluate a larger subset of patients with high ADA than the study by Porcel et al. Our study showed that hematologic malignancies were more prevalent than TBP in pleural effusion with ADA ≥ 150 IU/L, which is lower than the 250 IU/L suggested by a prior study. We further observed that a PMN count of less than 50% could serve as a robust indicator for hMPE. This observation is derived from the fact that the majority of patients presenting with very high ADA levels were diagnosed with PPE. On the other hand, pleural effusion with ADA above 70 IU/L was associated with an increased likelihood of TBP compared to ADA between 40 and 70 IU/L, which is consistent with previous studies [3, 6].

ADA should always be used in combination with other features such as lymphocyte proportion or LD/ADA. Combining ADA with lymphocyte proportion or LD/ADA, rather than using ADA alone, proved more helpful in diagnosing TBP, as demonstrated in our study. The pleural LD/ADA ratio, recently recognized as a significant marker in diagnosing TBP, was also found to be effective in our study [16, 26, 27]. Beukes et al. reported that LD/ADA was useful in distinguishing TBP when combined with ADA, but had no additional value when combined with lymphocyte proportion and ADA [28]. In contrast, our study, with a relatively large number of patients, demonstrated that combining LD/ADA with lymphocyte proportion was more accurate in diagnosing TBP than using lymphocyte proportion alone.

This study provided insight into the usefulness of lymphocyte proportion in the diagnosis of TBP as well as its cut-off. Traditionally, it was perceived that the pleural lymphocyte proportion in TBP was at least 50%. However, this conventional belief was contradicted by a study examining 382 patients with TBP, which found that 17% of patients had a pleural lymphocyte proportion below 50% [14]. Notwithstanding, literature regarding a lower limit of pleural lymphocytes for TBP remains scarce. Our study suggests that a proportion of ≥ 35% of pleural lymphocytes is a more accurate diagnostic threshold for TBP than the previously assumed ≥ 50%. Furthermore, our data provide valuable insights for clinicians, indicating that TBP is more probable when the pleural lymphocyte proportion exceeds 70%.

The primary strength of this investigation lies in its exclusive focus on routinely obtained diagnostic measures for pleural effusions, excluding CT findings or symptoms susceptible to inter-observer variability and recall bias. This approach enhances the applicability of our findings, especially in resource-limited settings. To the best of our knowledge, our report represents the largest study exploring hMPE and pleural effusions with high ADA. However, several limitations must be acknowledged. First, this study was retrospectively conducted in a single center with a large cancer center. Therefore, this study could have potential selection and information biases. Additionally, as the study was conducted in a region with an intermediate tuberculosis burden, the generalizability of our findings to other geographic areas remains uncertain. Hence, future international, multi-center prospective studies are warranted to validate our findings.

## Conclusions

In conclusion, the presence of hMPE should be considered in cases of pleural effusion with ADA ≥ 150 IU/L. This likelihood is further reinforced if coupled with a pleural PMN count of less than 50%, thrombocytopenia, or elevated serum LD levels. In pleural effusion with ADA levels ranging between 40 IU/L and 150 IU/L, a pleural lymphocyte proportion ≥ 35% and a pleural LD/ADA ratio < 18 are strong indicators of TBP.

### Electronic supplementary material

Below is the link to the electronic supplementary material.

Supplementary Material 1

## Data Availability

The datasets used and/or analysed during the current study are available from the corresponding author on reasonable request.

## Abbreviations

ADA: Adenosine deaminase
aOR: Adjusted odds ratio
CI: Confidence intervals
hMPE: Hemagologic malignant pleural effusion
IQR: Interquartile range
IU: International unit
LD: Lactate dehydrogenase
MPE: Malignant pleural effusion
NPV: Negative predictive values
PMN: Polymorphonuclear neutrophil
PPE: Parapneumonic effusion
PPV: Positive predictive values
sMPE: Solid malignant pleural effusion
TBP: Tuberculous pleurisy
WBC: White blood cells
