# Supplementary material for: Features which discriminate between tuberculosis and haematologic malignancy as the cause of pleural effusions with high adenosine deaminase
Source: Respir Res. 2024 Jan 4;25:17. doi: 10.1186/s12931-023-02645-6 (PMC10765929; doi:10.1186/s12931-023-02645-6)
Supplement: Supplementary file 1 — Supplementary Material 1 [file 12931_2023_2645_MOESM1_ESM.docx]

**Supplement Table 1.** Characteristics of pleural effusion with ADA ≥ 40 IU/L (n = 1134)

| Variables | Total  (n = 1134) | PPE  (n = 389) | TBP  (n = 375) | hMPE  (n = 85) | sMPE  (n = 177) | Others  (n = 108) |
| --- | --- | --- | --- | --- | --- | --- |
| Age, years | 60 (49–71) | 63 (53–71) | 58 (39–73) | 55 (36–66) | 61 (53–71) | 59 (51–68) |
| Male | 810 (71) | 316 (81) | 243 (65) | 57 (67) | 118 (67) | 76 (70) |
| Pleural effusion |  |  |  |  |  |  |
| PMN (%) | 24 (4–81) | 85 (73–92) | 6 (2–17) | 3 (1–12) | 19 (5–54) | 39 (8–78) |
| Lymphocytes (%) | 20 (5–63) | 4 (1–9) | 71 (50–84) | 21 (10–48) | 22 (8–48) | 12 (4–28) |
| Eosinophils (%) | 0.0 (0.0–0.0) | 0 (0–0) | 0 (0–0) | 0 (0–0) | 0 (0–0) | 0 (0–3) |
| Others (%) | 16 (8–34) | 9 (5–18) | 17 (10–27) | 69 (35–87) | 33 (16–57) | 18 (9–42) |
| pH | 7.3 (7.3–7.4) | 7.3 (7.2–7.4) | 7.3 (7.3–7.4) | 7.4 (7.3–7.4) | 7.4 (7.3–7.4) | 7.4 (7.3–7.4) |
| Glucose (mg/dL) | 78 (15–112) | 20 (3–89) | 95 (74–118) | 93 (47–115) | 59 (12–100) | 95 (49–131) |
| Protein (g/dL) | 4.6 (3.7–5.2) | 3.9 (3.0–4.8) | 5.0 (4.5–5.3) | 3.6 (2.9–4.6) | 5.0 (4.1–5.7) | 4.3 (3.4–5.1) |
| LD (IU/L) | 1440 (717–3743) | 3421 (1878–8035) | 671 (447–1023) | 1803 (1037–3519) | 2091 (944–4593) | 1456 (962–3056) |
| ADA (IU/L) | 69 (49–100) | 67 (48–121) | 84 (65–105) | 76 (53–171) | 51 (44–67) | 50 (44–63) |
| LD/ADA | 23 (9–55) | 53.0 (32.4–76.7) | 7.9 (5.6–11.4) | 17.8 (11.0–29.1) | 40.1 (18.9–73.2) | 28.4 (15.9–55.9) |
| Whole blood |  |  |  |  |  |  |
| WBC (×10³/μL) | 8.1 (5.7–12.3) | 12.4 (9.0–16.3) | 6.1 (5.1–7.7) | 6.8 (2.9–9.9) | 8.0 (6.0–11.8) | 8.2 (7.0–11.7) |
| Segmented neutrophils (%) | 74 (64–83) | 82 (76–87) | 68 (62–74) | 71 (53–81) | 72 (63–81) | 72 (62–83) |
| Platelets (×10³/μL) | 267 (178–346) | 265 (152–363) | 282 (227–347) | 139 (61–265) | 268 (180–347) | 254 (179–334) |
| Protein (g/dL) | 6.4 (5.7–7.0) | 5.9 (5.3–6.6) | 6.8 (6.2–7.2) | 5.6 (4.9–6.5) | 6.5 (6.1–7.1) | 6.4 (5.5–7.0) |
| LD (IU/ℓ) | 492 (396–648) | 488 (392–702) | 462 (391–546) | 1114 (612–1672) | 510 (389–664) | 529 (394–694) |
| Comorbidities |  |  |  |  |  |  |
| Solid tumor | 359 (32) | 140 (36) | 50 (13) | 2 (2) | 130 (73) | 37 (34) |
| Lung cancer | 183 (16) | 77 (20) | 12 (3) | 0 (0) | 80 (45) | 14 (13) |
| Others | 176 (16) | 63 (16) | 38 (10) | 2 (2) | 50 (28) | 23 (21) |
| Diabetes mellitus | 222 (20) | 104 (27) | 57 (15) | 13 (15) | 30 (17) | 18 (17) |
| History of tuberculosis | 101 (9) | 37 (10) | 37 (10) | 7 (8) | 9 (5) | 11 (10) |
| Hematologic malignancy | 87 (8) | 16 (4) | 15 (4) | 52 (61) | 0 (0) | 4 (4) |
| Heart failure | 43 (4) | 21 (5) | 7 (2) | 3 (4) | 3 (2) | 9 (8) |
| Liver cirrhosis | 43 (4) | 22 (6) | 15 (4) | 1 (1) | 3 (2) | 2 (2) |
| Renal replacement therapy | 31 (3) | 16 (4) | 6 (2) | 1 (1) | 1 (1) | 7 (6) |
| Connective tissue disease | 29 (3) | 10 (3) | 11 (3) | 0 (0) | 3 (2) | 5 (5) |
| Transplantation | 27 (2) | 10 (3) | 13 (3) | 1 (1) | 0 (0) | 3 (3) |

Data are reported as median (interquartile range) and number (%).

ADA = adenosine deaminase, hMPE = hematologic malignant pleural effusion, IU = international unit, LD = lactate dehydrogenase, PMN = polymorphonuclear neutrophil, PPE = parapneumonic effusion, sMPE = solid malignant pleural effusion, TBP = tuberculous pleurisy, WBC = white blood cells.

**Supplement Table 2.** Comparison of characteristics of hematologic malignant pleural effusion

| Variables | Hematologic MPE  (n = 85) | Lymphoma  (n = 70) | Leukemia & multiple myeloma  (n =15) | *P* value |
| --- | --- | --- | --- | --- |
| Age, years | 55 (36–66) | 50 (31–66) | 62 (59–66) | 0.069 |
| Male | 57 (67) | 48 (69) | 9 (60) | 0.554 |
| Pleural effusion |  |  |  |  |
| PMN (%) | 3 (1–13) | 3 (1–11) | 3 (1–11) | 0.435 |
| Lymphocytes (%) | 21 (10–48) | 24 (11–55) | 5 (3–55) | 0.001 |
| Eosinophils (%) | 0 (0–0) | 0 (0–0) | 0 (0–0) | 0.635 |
| Others (%) | 69 (35–87) | 60 (30–84) | 80 (58–95) | 0.032 |
| pH | 7.4 (7.3–7.4) | 7.4 (7.3–7.4) | 7.4 (7.2–7.4) | 0.661 |
| Glucose (mg/dL) | 93 (47–115) | 84 (42–112) | 119 (90–149) | 0.026 |
| Protein (g/dL) | 3.6 (2.9–4.6) | 3.6 (2.9–4.3) | 4.2 (2.7–4.3) | 0.596 |
| LD (IU/L) | 1803 (1037–3519) | 1815 (1061–4119) | 1294 (903–4119) | 0.254 |
| ADA (IU/L) | 76 (53–171) | 94 (57–179) | 52 (44–179) | 0.010 |
| LD/ADA | 17.8 (11.0–29.1) | 17.1 (10.4–26.6) | 20.0 (14.4–26.6) | 0.327 |
| Whole blood |  |  |  |  |
| WBC (×10³/μL) | 6.8 (2.9–9.9) | 6.9 (2.9–9.8) | 6.6 (2.9–9.8) | 0.995 |
| Segmented neutrophils (%) | 71 (53–81) | 73 (59–81) | 57 (17–81) | 0.076 |
| Platelets (×10³/μL) | 139 (61–265) | 162 (64–274) | 82 (52–274) | 0.083 |
| Protein (g/dL) | 5.6 (4.9–6.5) | 5.6 (4.9–6.4) | 5.6 (4.8–6.4) | 0.744 |
| LD (IU/ℓ) | 1114 (612–1672) | 1114 (615–1695) | 1155 (491–1512) | 0.500 |
| Comorbidities |  |  |  |  |
| Solid tumor | 2 (2) | 1 (1) | 1 (7) | 0.324 |
| Diabetes mellitus | 13 (15) | 6 (9) | 7 (47) | 0.001 |
| History of tuberculosis | 7 (8) | 6 (9) | 1 (7) | 1.000 |
| Hematologic malignancy | 52 (61) | 42 (60) | 10 (67) | 0.773 |
| Heart failure | 3 (4) | 3 (4) | 0 (0) | 1.000 |
| Liver cirrhosis | 1 (1) | 1 (1) | 0 (0) | 1.000 |
| Renal replacement therapy | 1 (1) | 0 (0) | 1 (7) | 0.176 |
| Connective tissue disease | 0 (0) | 0 (0) | 0 (0) | - |
| Transplantation | 1 (1) | 1 (1) | 0 (0) | 1.000 |

Data are reported as median (interquartile range) and number (%).

ADA = adenosine deaminase, IU = international unit, LD = lactate dehydrogenase, MPE = malignant pleural effusion, PMN = polymorphonuclear neutrophil, WBC = white blood cells.

**Supplement Table 3**. Univariable and multivariable analyses with logistic regression models for variables associated with PPE in pleural effusion with ADA ≥ 40 IU/L

| Variables | Univariable | | Multivariable | |
| --- | --- | --- | --- | --- |
|  | **OR (CI 95%)** | ***P* value** | **Adjusted OR (CI 95%)** | ***P* value** |
| Age ≥ 65 years | 1.25 (0.98–1.61) | 0.077 |  |  |
| Pleural effusion |  |  |  |  |
| ADA, IU/L |  |  |  |  |
| 40 ≤ ADA < 70 | Reference |  | Reference |  |
| 70 ≤ ADA < 150 | 0.59 (0.45–0.78) | <0.001 | 1.40 (0.85–2.29) | 0.187 |
| ADA ≥ 150 | 3.29 (2.19–4.94) | <0.001 | 2.43 (1.28–4.63) | 0.007 |
| PMN ≥ 50% | 29.41 (20.77–41.65) | <0.001 | 10.20 (6.60–15.75) | <0.001 |
| Eosinophils ≥ 10% | 0.38 (0.17–0.87) | 0.022 |  |  |
| pH < 7.2 | 5.05 (3.22–7.91) | <0.001 | 3.18 (1.54–6.59) | 0.002 |
| Glucose < 60 mg/dL | 5.22 (3.98–6.83) | <0.001 | 1.70 (1.11–2.60) | 0.015 |
| Protein ≥ 2/3× serum ULN | 0.32 (0.23–0.46) | <0.001 | 0.36 (0.21–0.62) | <0.001 |
| LD/ADA ≥ 18 | 14.66 (10.17–21.13) | <0.001 | 4.16 (2.43–7.12) | <0.001 |
| Serum |  |  |  |  |
| WBC ≥ 10,000/μL | 8.44 (6.35–11.21) | <0.001 | 3.45 (2.30–5.18) | <0.001 |
| Platelets < 150,000/μL | 0.78 (0.59–1.04) | 0.087 |  |  |
| Protein < serum LLN | 0.32 (0.24–0.42) | <0.001 | 0.55 (0.36–0.83) | 0.005 |
| LD ≥ 2× serum ULN | 0.77 (0.57–1.03) | 0.076 |  |  |

To avoid multicollinearity, this analysis included a proportion of pleural PMN (%) only without that of pleural lymphocytes.

ADA = adenosine deaminase, CI = confidence interval, IU = international unit, LD = lactate dehydrogenase, LLN = lower limit of normal, OR = odds ratio, PMN = polymorphonuclear neutrophil, PPE = parapneumonic effusion, ULN = upper limit of normal, WBC = white blood cells.

**Supplement Table 4**. Univariable and multivariable analyses with logistic regression models for variables associated with solid malignant pleural effusion in pleural effusion with ADA ≥ 40 IU/L

| Variables | Univariable | | Multivariable | |
| --- | --- | --- | --- | --- |
|  | **OR (CI 95%)** | ***P* value** | **Adjusted OR (CI 95%)** | ***P* value** |
| Age ≥ 65 years | 0.99 (0.71–1.37) | 0.935 |  |  |
| Pleural effusion |  |  |  |  |
| ADA, IU/L |  |  |  |  |
| 40 ≤ ADA < 70 | Reference |  | Reference |  |
| 70 ≤ ADA < 150 | 0.27 (0.18–0.41) | <0.001 | 0.24 (0.15–0.39) | <0.001 |
| ADA ≥ 150 | 0.14 (0.05–0.34) | <0.001 | 0.10 (0.03–0.28) | <0.001 |
| Lymphocytes, % |  |  |  |  |
| Lymphocytes < 35 | Reference |  | Reference |  |
| 35 ≤ Lymphocytes < 70 | 1.38 (0.95–2.02) | 0.093 | 2.43 (1.45–4.06) | 0.001 |
| Lymphocytes ≥ 70 | 0.43 (0.26–0.72) | 0.001 | 1.07 (0.54–2.12) | 0.850 |
| Eosinophils ≥ 10% | 0.74 (0.29–1.92) | 0.541 |  |  |
| pH < 7.2 | 0.27 (0.11–0.67) | 0.005 |  |  |
| Glucose < 60 mg/dL | 1.73 (1.25–2.41) | 0.001 | 2.03 (1.32–3.13) | 0.001 |
| Protein ≥ 2/3× serum ULN | 3.08 (2.19–4.33) | <0.001 | 4.13 (2.72–6.27) | <0.001 |
| LD/ADA ≥ 18 | 2.99 (2.07–4.34) | <0.001 | 2.81 (1.63–4.85) | <0.001 |
| Serum |  |  |  |  |
| WBC ≥ 10,000/μL | 0.87 (0.61–1.24) | 0.451 | 0.62 (0.41–0.94) | 0.025 |
| Platelets < 150,000/μL | 0.77 (0.50–1.21) | 0.256 |  |  |
| Protein < serum LLN | 1.97 (1.34–2.91) | 0.001 |  |  |
| LD ≥ 2× serum ULN | 1.23 (0.83–1.81) | 0.297 |  |  |

To avoid multicollinearity, this analysis included a proportion of pleural lymphocytes (%) only without that of pleural polymorphonuclear neutrophils.

ADA = adenosine deaminase, CI = confidence interval, IU = international unit, LD = lactate dehydrogenase, LLN = lower limit of normal, OR = odds ratio, PMN = polymorphonuclear neutrophil, ULN = upper limit of normal, WBC = white blood cells.
